# Cannabidiol (CBD) in Cancer Management

**DOI:** 10.3390/cancers14040885

**Published:** 2022-02-10

**Authors:** Kylie O’Brien

**Affiliations:** 1 Adelaide Campus, Torrens University, Adelaide, SA 5000, Australia; satorimedicine@gmail.com; 2 NICM Health Research Centre, Western Sydney University, Westmead, Sydney, NSW 2145, Australia; 3 Releaf Group Ltd., St Kilda, VIC 3182, Australia; 4 International College of Cannabinoid Medicine, iccm.co, London N1 7GU, UK

**Keywords:** cannabidiol, cannabis, endocannabinoid system, cancer, tumours

## Abstract

**Simple Summary:**

Cannabidiol (CBD) is one of the main constituents of the plant Cannabis sativa. Surveys suggest that medicinal cannabis is popular amongst people diagnosed with cancer. CBD is one of the key constituents of cannabis, and does not have the potentially intoxicating effects that tetrahydrocannabinol (THC), the other key phytocannabinoid has. Research indicates the CBD may have potential for the treatment of cancer, including the symptoms and signs associated with cancer and its treatment. Preclinical research suggests CBD may address many of the pathways involved in the pathogenesis of cancers. Preclinical and clinical research also suggests some evidence of efficacy, alone or in some cases in conjunction with tetrahydrocannabinol (THC, the other key phytocannabinoid in cannabis), in treating cancer-associated pain, anxiety and depression, sleep problems, nausea and vomiting, and oral mucositis that are associated with cancer and/or its treatment. Studies also suggest that CBD may enhance orthodox treatments with chemotherapeutic agents and radiation therapy and protect against neural and organ damage. CBD shows promise as part of an integrative approach to the management of cancer.

**Abstract:**

The plant Cannabis sativa has been in use medicinally for several thousand years. It has over 540 metabolites thought to be responsible for its therapeutic effects. Two of the key phytocannabinoids are cannabidiol (CBD) and tetrahydrocannabinol (THC). Unlike THC, CBD does not have potentially intoxicating effects. Preclinical and clinical research indicates that CBD has a wide range of therapeutic effects, and many of them are relevant to the management of cancer. In this article, we explore some of the potential mechanisms of action of CBD in cancer, and evidence of its efficacy in the integrative management of cancer including the side effects associated with its treatment, demonstrating its potential for integration with orthodox cancer care.

## 1. Introduction

Survey data indicates that cancer sufferers are using cannabis medicinally. A cross-sectional survey in 926 patients at the Fred Hutchinson Cancer Research Centre (Seattle) found that 66% had used cannabis previously, with 24% of respondents having used cannabis in the past year and 21% in the past month. Of the 24% (n = 222) of respondents who were active users, around 75% used cannabis for physical symptoms (pain, nausea, appetite), 63% for neuropsychiatric symptoms (stress, coping with illness, depression/improve mood, sleep), and 26% reported they believed cannabis was helping to treat their cancer. Encouragingly, regardless of symptom, approximately 51% judged cannabis to be of ‘major benefit’ and 39% of ‘moderate benefit’ [1]. An anonymous online survey of 612 US-based members of the Breastcancer.org and Healthline.com communities with a self-reported diagnosis of breast cancer within 5 years found that 42% used cannabis for relief of symptoms (including pain (78%), insomnia (70%), anxiety (57%), stress (51%) and nausea/vomiting (46%)) with 46% of the belief that cannabis can treat the cancer itself. Of those using cannabis, 79% had used it during treatment (systemic therapies, radiation, surgery) [2]. 

The plant Cannabis sativa L. has several hundred secondary metabolites and cannabidiol (CBD) is one of the key phytocannabinoids. In this paper, I will explore what evidence exists that CBD may be useful in the integrative management of cancer, including some of its relevant mechanisms of action, evidence of efficacy in the treatment of cancer, and symptoms associated with cancer and/or its treatment and evidence that CBD may enhance orthodox cancer treatments. Note that there are several good review papers which I would draw readers’ attention to, including Seltzer and colleagues [3]; Mangal and colleagues [4]; and Moreno and colleagues [5]. In particular, Seltzer and colleagues’ paper [3] provides an excellent and in-depth summary of mechanisms of action of CBD in various forms of cancer, illustrating the extensiveness of the preclinical research that supports the contention that CBD is an efficacious anti-cancer agent.

However, before delving into the scientific evidence on CBD and cancer, it is important to understand a bit of the basics, about the plant Cannabis sativa and our endocannabinoid system.

## 2. What Is Cannabis and Cannabidiol?

The plant Cannabis sativa has been used medicinally for thousands of years in many cultures including Chinese, Japanese, Indian, and Egyptian, whilst its medicinal use in western countries such as the US, England, and parts of Europe began to occur much later, particularly in the 19th century [6,7,8,9,10,11].

### 2.1. Constituents of Cannabis

There are over 540 secondary metabolites in the cannabis plant, of which there are over 120 phytocannabinoids, divided into 11 classes [7,12,13]. Tetrahydrocannabinol (THC) and cannabidiol (CBD) are the two most well-researched of the phytocannabinoids. In addition, over 200 terpenes have been isolated from cannabis, along with phenols, steroids, polysaccharides, coumarins, glycosides, flavonoids, alcohols, and other plant nutrients, and these have their own therapeutic actions [13,14]. The so-called ‘entourage effect’ refers to the cooperative effect between the various constituents of the plant, whereby the therapeutic effect of the other constituents may contribute to the overall therapeutic effect of the main phytocannabinoids (i.e., THC, CBD). I like to use the analogy of a rock band, with the rockstars on stage being THC and CBD, the rest of the band members the other phytocannabinoids and terpenes, and the ‘roadies’ being other plant nutrients like polyphenols and so on—there’s no show without the band or the roadies. 

### 2.2. Cultivars of Cannabis

There are several hundred different ‘strains’ or cultivated varieties (‘cultivars’) of cannabis, and their chemical profiles will differ. That is, the relative amounts of key phytocannnabinoids, terpenes, and other plant nutrients will be different in different cultivars of cannabis. And so, the therapeutic actions of different cultivars can also differ. 

### 2.3. Cannabis and Cannabidiol Products

Medicinal cannabis products include dried flower (which can be smoked or vaped) and proprietary forms: (1) cannabis-based liquid extracts, e.g., nabiximols (approximately 1:1 ratio of THC and CBD); (2) phytocannabinoid botanicals: dense cannabis extracts manufactured as oils, oils in capsules, pills, sublingual or intranasal sprays, suppositories, transdermal patches, E-Liquids for vaporization, and topical ointments; and (3) single molecule drugs: synthetic or semi-synthetic prescription drugs (e.g., nabilone, dronabinol, which are FDA-approved) [15]. 

Note that in the consumer literature, the terms *full-spectrum* and *broad-spectrum* are often used in relation to cannabis and CBD products: full-spectrum denotes the presence of all the phytocannabinoids, terpenes, and other plant nutrients naturally found in the plant in the final product, and broad-spectrum denotes the presence of many of the phytocannabinoids and terpenes naturally found in the plant but not all of them. Typically, a *broad-spectrum* CBD product will have the THC removed [16]. 

Flower and cannabis oil products differ in terms of their relative amounts of the key phytocannabinoids, THC and CBD, as well as the types and relative amounts of terpenes and minor phytocannabinoids (which have their own therapeutic actions) [14]. 

If we consider CBD products available on the market, it is clear that they vary considerably. Whole plant (full-spectrum, broad-spectrum) CBD products will differ in terms of amount (concentration, percentage) of CBD and other phytocannabinoids present, as well as the types and relative amounts of terpenes and other plant nutrients present. CBD-predominant products typically have very low amounts of THC. If the CBD oil has been derived from a hemp plant (hemp is simply a cannabis cultivar bred to have a very low amount of THC), then it will contain less than 0.3% THC if produced in the US (the upper legal limit of THC). An important point to note is that whole plant products are likely to work differently to CBD isolate. 

### 2.4. Differences between THC and CBD

THC is responsible for the potentially intoxicating effects associated with cannabis (the potential for causing intoxication, i.e., the euphoria or ‘high’ associated with cannabis, is dose-dependent), but unlike THC, CBD is not potentially intoxicating and not associated with the typical symptoms associated with cannabis intoxication [13,17], making it perhaps more attractive as a treatment option. Both THC and CBD have many therapeutic actions in common, but their mechanisms of action differ [9]. THC is a partial agonist of the CB1 and CB2 receptors, similar to AEA [18]. However, CBD has a low affinity to the cannabinoid receptors, and is believed to exert its actions predominantly via activating the ECS indirectly, as well as interacting with other targets or receptors [19,20,21,22]

### 2.5. Therapeutic Actions of CBD

CBD has many therapeutic actions, set out in Table 1. From this table, we see how broad the therapeutic actions of this phytocannabinoid are, and we already start to see the potential relevance of CBD to cancer, its pathomechanisms, and signs/symptoms associated with cancer and its orthodox treatment. I will discuss CBD’s actions in relation to cancer in more detail shortly.

Now that we understand a little about CBD, let’s look at our endocannabinoid system (ECS). As in many respects, the reason that cannabis may be so broad in its therapeutic applications is due to the presence of this ‘ready-made system’ that the constituents of cannabis interact with. I will then look at what happens to our ECS in cancer, and how CBD may be able to assist. 

## 3. Our Endocannabinoid System

### 3.1. Role of the Endocannabinoid System

The ECS is one of the most important neuroregulatory systems we have, responsible for the homeostasis of most systems in the body. The ECS modulates the following: the immune system (innate, adaptive); inflammation; pain/analgesia; our stress response, emotions/moods, cognitive function, memory and memory extinction; sleep; gastrointestinal (GI) tract homeostasis (including regulation of food intake and satiation, gastroprotection, nausea and emesis, gastric secretion, visceral sensation, GI motility, ion transport, intestinal inflammation and cell proliferation in the gut); energy homeostasis and regulation of lipid and glucose metabolism; embryological development; the cycle of cell life and death, cancer cell control, cyto-protection; neurotransmitters, neuroprotection, neural plasticity, and many others [9,15,20,28,29,30,31,32,33]. 

### 3.2. Components of the Endocannabinoid System

Discovered in the 1990′s, at a simplistic level there are three key components of the endocannabinoid system (ECS): (1) lipid-derived endocannabinoids (the two main ones are N-arachidonylethanolamine or anandamide (abbreviated AEA] and 2-arachidonylglycerol (2-AG)), but there are others), the enzymes that synthesise and degrade them (fatty acid amide hydrolase (FAAH] and mono acyl glycerol lipase (MAGL) being two main ones degrading AEA and 2-AG respectively) plus various transporter systems, and (2) cannabinoid receptors (CB1 and CB2 receptors) [15]. 

However, it is much more complex and what we have are many more components that make up an ‘extended ECS’ [9]. Firstly, there are other receptors that cannabinoids (endocannabinoids and/or phytocannabinoids) interact with, including G-Protein Receptors (GPR55, GPR18, and GPR119), transient receptor potential vanilloid [TRPV] ion channels (TRPV1 and 2), and peroxisome proliferator activated receptors (PPARα and PPARϒ) [5,10,34]. There are other endocannabinoid-like substances (e.g., N-palmitoylethanolamide [PEA], oleoylethanolamide [OEA] and oleamide). There are also more recently discovered hemopressin-derived peptides (inverse agonists of CB1 receptors), novel lipid compounds (lipoxins and resolvins) that also regulate physiological allostasis, and n-3 endocannabinoid epoxides originating from docosahexanoic acid (DHA) and eicosapentanoic acid (EPA) (de Melo Reis et al., 2021). 

Cannabimimetic compounds including omega (n-3 and n-6) fatty acids can signal through the ECS (Frietas et al., 2017); indeed, both AEA and 2-AG are derived from arachidonic acid (from n-6 PUFAs) and levels of the endocannabinoids and their activity are influenced by the ratio of n-6 and n-3 polyunsaturated fatty acids in our diet [35]. 

The endocannabinoids actually have several biosynthetic and degrading pathways and enzymes which may be shared with endocannabinoid-like mediators; degradation of AEA and 2-AGA leads to arachidonic acid plus several other bioactive signalling molecules [36,37,38,39]. The term ‘endocannabinoidome’ was coined to describe the endocannabinoids, endocannabinoid-like mediators, and the many receptors and metabolic enzymes [36]. See de Melo Reis et al. [35] and Di Marzo and Piscitelli [36] for good descriptions of the ECS and regulation of homeostasis.

### 3.3. Where Are the CB1 Receptors and CB2 Receptors Located?

CB1 receptors are abundant in the central nervous system (brain, spinal cord) but are also found peripherally in many tissues and organs (though at a lower level of expression than in the brain) [40]. Several isoforms have been found: CB1, CB1A, and CB1B [41,42]. 

CB1 receptors are found in high concentrations in areas of the brain associated with mood/emotions and cognitive processes as well as movement [10]. Another interesting fact is that CB1 receptors appear ten times more frequently in the brain then mu-opioid receptors, and can co-localise with them to augment the pain-relieving effects of opioids [43,44]. CB1 receptors maintain the delicate balance between neuronal inhibition and excitation, in particular in GABAergic, glutamatergic, and dopaminergic transmission [45]. CB1 receptors are also abundant on the outer membranes of mitochondria [46]. 

CB2 receptors are particularly abundant in the cells and tissues and organs of the immune system, are also found in many other parts of the body including the brain (where they are highly inducible under conditions of inflammation) [9,47]. CB2 receptors are key mediators of cannabinoid regulation of the immune and inflammatory systems [15], where in general, CB2 receptor activation usually mediates immunosuppressive effects, attenuating the autoimmune inflammatory response, and thereby limiting tissue injury [48]. In addition, a CB2 isoform has been identified, CB2A, in the liver, spleen, neurons, and brain cortex [49]. See Table 2 for locations of CB1 and CB2 receptors in the body.

### 3.4. How Does the Endocannabinoid System Work?

AEA is a partial agonist at CB1 and CB2 receptors, whilst 2-AG is a full agonist [40,45]. CB1 and CB2 receptors are G-protein-coupled receptors, and when activated signal through fast pathways (i.e., Ca^2+^ and K^+^ currents) and/or slow pathways (e.g., cyclic AMP-protein kinase A and others) [35]. At a simplistic level, in the nervous system the ECS functions as a retrograde signalling system, decreasing the release and transmission of neurotransmitters. 

In the nervous system, endocannabinoids are synthesised on demand from plasma membrane phospholipids in the post-synaptic neuron in response to increased intracellular calcium concentration and/or activated G-coupled receptors [10,83]. When synthesis is triggered, the endocannabinoids move in a retrograde fashion across the synaptic space, from post-synaptic to the presynaptic region, binding with cannabinoid receptors on the presynaptic neuron, and leading to suppression of neuronal excitation and inhibition of depolarisation-induced neurotransmitter release [10]. What happens downstream depends on whether the neurotransmitter is excitatory (e.g., glutamate) or inhibitory (e.g., Υ-aminobutyric acid, GABA) [10,40]. The endocannabinoids are then degraded by their respective enzymatic pathways [39]. 

CB1 and CB2 receptors can activate many different intracellular signal transduction pathways, including (depending on cell type): protein kinase A, protein kinase C, Raf-1, JNK, mitogen-activated protein kinases (MAPK), p38 MAPKs, extracellular signal-regulated kinase (ERK 1, 2), c-fos, c-jun, phosphoinositide 3-kinase (PI3K)/protein kinase B (Akt) pathways, mammalian target of rapamycin (mTOR) and more [83,84,85]. Depending on the ligand and subcellular environment, the eventual outcome could be promotion of cell survival or cell death [83]. 

That is the simple explanation, but of course it is much more complex than that given the existence of other receptors that the endocannabinoids can bind with and the existence of endocannabinoid-like substances. In addition, degradation of 2-AG also produces bioactive signalling molecules, some of which have opposing effects [37,86]. For an in-depth exploration of the ECS, see de Melo Reis and colleagues [35].

Now that we understand something of the ECS, we will now look at the herb Cannabis sativa and how it interacts with the components of our extended ECS.

## 4. The Endocannabinoid System in Cancer

Cannabinoid receptors are widely expressed on cancer cells as well as normal cells [5]. Research indicates that ECS dysfunction is part of the pathomechanism of many diseases, including cancer (Moreno et al., 2020) and those signs and symptoms associated with cancer and its treatment, such as anxiety, depression, poor sleep [9], and so on. 

Cannabinoid receptor stimulation can lead to different outcomes, with protective effects in some tumour subtypes and unfavourable effects in others [5]. Cannabinoid receptors and other receptor members of the extended ECS have been found to be over-expressed or under-expressed in various tumours [4,5]. For example, TRPV1 is under-expressed in glioblastoma multiforme but over-expressed in lung adenocarcinoma; CNR1 (gene coding for CB1 receptors) is under-expressed in lung adenocarcinoma, thyroid carcinoma, breast invasive carcinoma, and uterine corpus endometrial carcinoma and over-expressed in cholangiocarcinoma; CB2 receptors are overexpressed in HR+ breast cancer and gliomas [5,87,88]. Overexpression of CB1 and CB2 receptors was found to be correlated with poor prognosis in stage 4 colorectal cancer [89,90]. 

Moreno and colleagues [5] explain that changes in expression and activation of cannabinoid receptors and their capacity to form functional heteromers with other receptors alter a cell’s tumorigenic potential and signalling properties. For example, human epidermal growth factor receptor 2 (HER2) forms heteromer complexes with the CB2 receptor in breast cancer cells, and the expression of such complexes correlates with poor prognosis [91]. However, the disruption of these heteromer complexes promotes anti-tumour responses and may represent a new therapeutic target. THC has been found to disrupt HER2–CB2 receptor complexes by selectively binding to CB2 receptors, hampering HER2 activation (by interfering with its homodimerization), and impairing HER+ breast cancer cell viability [91]. Other research has found that CB2 receptors and GPR55, both of which are elevated in most tumours and control cancer cell fate, form heteromers in cancer cells and that targeting these heteromers modulates cancer cell signalling (Moreno et al., 2014). Experiments have shown these heteromers to have unique pharmacological and signalling properties, displaying cross-talk and cross-antagonism at the level of cAMP and p-ERK-1/2 pathways. Further experiments demonstrated an antagonistic effect of THC on GPR55-modulated CB2 receptor signalling via these CB2 receptor-GPR55 heteromers [92]. 

Some evidence suggests AEA can inhibit proliferation, migration, and invasiveness (in vitro and in vivo studies) and directly inhibit angiogenesis. In an experiment using a proangiogenic phenotype of the highly invasive and metastatic breast cancer cells (MDA-MB-231), an AEA analogue was found to inhibit all the pro-angiogenic factors produced by these cells and consequently these cells lost their ability to stimulate endothelial cell proliferation in vitro [93]. However, some research in gliomas suggests that AEA levels are increased, suggesting pro-cancer activity [94] and in colorectal cancer, depending on the state of the cancer, endocannabinoids can either inhibit or promote CRC growth [3]. In gliomas, the direction of AEA levels has not been consistent: some research has found lower levels of AEA compared with non-tumour tissue, but other studies have found higher levels in gliomas and also in meningiomas, whilst 2-AG has been found to be upregulated in both types of brain cancer [5,95,96,97]. 

Mangal and colleagues [4] explain that the role of the ECS is specific to the type of cancer and remind us that cancer is a heterogenous disease. Thus, we cannot assume changes in the ECS are going to be the same in all cancers. Also, given that the ECS is a homeostatic system, it begs the question whether raised levels of endocannabinoids is part of the pathogenesis or a response from the body to bring it back into balance?

## 5. Anti-Cancer Mechanisms of Action of CBD

Various preclinical studies, from cancel cell line studies to rodent models of cancer, have revealed that various cannabinoids (including endocannabinoids AEA, 2-AG, phytocannabinoids THC, CBD, and synthetic cannabinoid receptor agonists) have anti-cancer activity, addressing many of the ‘hallmarks of cancer’ [98]. 

Pro-apoptotic, anti-proliferative actions of cannabinoids have been demonstrated in many types of cancers [3,5,99]. Cannabinoid actions include cell cycle arrest, induction of apoptosis, inhibition of chemotaxis, cancer cell migration, adhesion, angiogenesis, invasion, and metastasis [3,100,101,102,103]. Yet, in general, the viability of normal (non-transformed) cells appears to be unaffected or even favoured under certain conditions by cannabinoids (though there are some exceptions); the stimulation of cannabinoid receptors appears to activate different signalling mechanisms in transformed and normal cells [98]. 

Considering CBD specifically, the literature indicates that in many animal cancer models, CBD’s ability to inhibit the progression of different types of cancer has been demonstrated, including in glioblastoma (GBM), breast, lung, prostate and colon cancer, and melanoma [104,105]. For example, in a mice model of melanoma, CBD treatment was associated with a significant reduction in tumour size compared with placebo, and increased survival [105]. 

It is apparent that CBD affects many tumoral features and molecular pathways [106] and perhaps this is not surprising, given the fact that CBD has many targets. Much of CBD’s anti-tumour activity is via its regulation of reactive oxygen species (ROS), endoplasmic reticulum (ER) stress, and immune modulation (all important in tumorigenesis) [3]. For example, although CBD has potent antioxidant activity, CBD has been found to be cytotoxic to human glioma cells, triggering caspase activation and oxidative stress [107]. CBD exposure to human glioma cells caused an early production of ROS, depletion of intracellular glutathione, and increased activity of glutathione reductase and glutathione peroxidase, but it did not impair primary normal glia. A different sensitivity to the anti-proliferative effect of CBD in glioma cells and non-transformed cells was demonstrated, believed to be associated with a selective ability of CBD to induce ROS production and activate caspase in tumour cells [107]. Similarly, other research has found that exposure of non-malignant brain cells (including human neural stem/progenitor cells and immortalized human foetal astrocytes) to CBD was not linked with induction of apoptosis [98,108]. Other experiments in glioma [109], leukemia (e.g., [110]), and breast cancer cells [103] have also demonstrated that CBD triggers a signalling mechanism that involves the generation of ROS [103]. 

Glioma cell research demonstrates CBD alone or in conjunction with other agents can induce cell death, inhibit cell migration and invasion, reduce size of tumours, reduce vascularisation, and induce tumour regression and increased survival [3]. Earlier in-vivo research found that CBD could impair migration of U87 glioma cells, but the mechanism did not appear to be via the classic cannabinoid receptors or receptors coupled to Gi/o proteins [111]. Seltzer and colleagues [3] reported that apoptosis induced by CBD in gliomas appeared independent of cannabinoid receptors but dependent on TRPV2. CBD has been found to activate TRPV2, decreasing proliferation and increasing susceptibility to drug-induced cell death in human cancer cells [112]. In an in-vivo experiment using glioma tumour tissues excised from nude mice, CBD was found to exert antitumoral effects via decreasing the activity and content of 5-lipoxygenase (LOX) and its end-product leukotriene, though no effect was found on COX-2 and end-product prostaglandin E_2_ (both 5-LOX and COX-2 are isoenzymes very involved in the control of cell growth and death in the CNS) [94]. Other glioma research has shown that CBD inhibits U87-MG and T98G glioma cell proliferation and invasiveness, and can downregulate ERK and Akt pro-survival signalling pathways and decrease hypoxia inducible factor HIF-1α expression in U87-MG cells (HIF-1α is a critical regulator of the hypoxic response, upregulating cell survival associated molecules, promoting invasion and tumour angiogenesis and the switch to glycolytic metabolism) [106]. 

In very recent research, according to Khodadadi and colleagues [113], the tumour microenvironment and its interaction with tumour cells is critically involved in the development, progression, and resilience of glioblastoma (Khodadadi et al., 2021). They argue that interactions between angiogenic and immune factors are determining factors in tumour vascularisation, immune profile and the lack of responsiveness to the immune system that characterises glioblastoma. In their research using a mice model of glioblastoma (using modified glioblastoma cells from humans), inhalation of CBD for seven days was found to significantly impact the cellular and molecular signalling of the tumour microenvironment [113]. Inhaled CBD limited tumour growth and altered the dynamics of the tumour microenvironment: it repressed P-selectin (which, in cancer, helps tumours metastasise and become treatment resistant), apelin (elevated in glioblastoma, acting to support blood vessel growth and promote cancer stem cells), and interleukin (IL)-8 (typically secreted by glioblastomas to promote cell migration and angiogenesis and found to be elevated in many forms of cancer) [113]. CBD blocked a key immune checkpoint, indoleamine 2,3-dioxygenase (IDO), which functions to block the immune response in tumours. It also enhanced the expression of a complex that aids the immune system to recognise cancer, the cluster of differentiation (CD)103 indicating improved antigen presentation, increased CD8 responses (a protein that aids the immune response), and decreased innate lymphoid cells within the tumour [113].

In breast cancer, CBD exerts anti-proliferative effects through many mechanisms including apoptosis, autophagy, and cell cycle arrest [3,114,115]. CBD has been found to induce programmed cell death in breast cancer cells via coordinating cross-talk between apoptosis and autophagy, in a manner independent of cannabinoid and vanilloid receptors [103]. CBD was also found to downregulate ID1, a regulator of metastasis in breast cancer cell lines [116]. Another experiment in rats which demonstrated anti-tumour properties of five phytocannabinoids (CBD, THC, cannabigerol, cannabichromene, cannabidiol acid) found that CBD most strongly inhibited breast cancer cell growth [114]. CBD and a CBD-enriched extract inhibited breast xenograft tumours in rodents and reduced lung metastases in rodents. From their various experiments, they proposed that CBD does not have a unique mode of action in the cell lines they examined, but found that in MDA-MB-231 breast cancer cells, CBD induced apoptosis via direct and indirect activation of CB2 receptors and TRPV1 receptors and via cannabinoid and vanilloid receptor-independent elevation of intercellular Ca^2+^ and ROS [114].

CBD can modulate the tumour microenvironment, reducing secretion of cytokines from cancer cells. Decreased recruitment of macrophages from the tumour microenvironment by cancer cells suppresses angiogenesis within the tumour, limiting the supply of oxygen and nutrients needed for tumour growth [117]. CBD can inhibit exosomes and microvesicles (EMV), mediators of intercellular communication released by cells which affect many physiological and pathological processes including cell migration, differentiation, and angiogenesis. Increased EMV release has been found in cancer, in particular in association with chemotherapy resistance and in the active transfer of pro-oncogenic factors, and chemotherapeutic drug resistance may be partly due to EMV shedding from cancer cells which aids increased active drug efflux [25]. CBD has been found to significantly and dose-dependently inhibit the release of EMVs in three cancer cell lines: prostate cancer (PC3), hepatocellular carcinoma (HEPG2), and breast adenocarcinoma (MDA-MB-231). The mechanism of action may be associated with changes in mitochondrial function (specifically modulation of STAT3 and prohibitin expression) [25]. 

Other effects of CBD include inhibition of GPR55, known to be elevated in several cancers such as aggressive triple negative breast cancer, where elevated levels are associated with higher chance of developing metastases [118]. GPR55 is related directly or indirectly with changes that promote malignant growth including uncontrolled cancer cell proliferation, angiogenesis, cancer cell adhesion, cancer cell migration, and metastasis [118,119]. CBD was shown to significantly decrease adhesion to endothelial cells and migration of HCT116 cells (metastatic colon cancer cell line), an inhibitory effect that was prevented by GPR55 siRNA knock down in cancer cells [119]. Increased GPR55 has been found in human pancreatic ductal adenocarcinoma (PDAC) specimens [120]. In a mice model of PDAC, pharmacological blockade of GPR55 with CBD, gemcitabine, and CBD plus gemcitabine increased the rodent lifespan compared to vehicle (mean survival 25.4 days, 27.8 days, 52.7 days, and 18.6 days respectively), with many of the signalling pathways involved in reducing PDAC cell cycle progression and cell growth identified [120].

Note that animal research typically uses isolates of CBD (and THC). More research is needed investigating the effects of whole plant (full-spectrum) forms of CBD medicines, including those that compare isolate to whole plant CBD medicines in all forms of cannabis research, as it is likely that isolates will behave differently to full-spectrum CBD medicines. Gallily and colleagues [121] found a very different dose-response curve when investigating the anti-nociceptive and anti-inflammatory effects of CBD in a rodent model: the shape of the dose-response curves changed from bell-shaped (anti-pain effect) or U-shaped (anti-inflammatory effect on paw swelling) for CBD isolate to linear (i.e., an increasing response with increasing dosage, reducing zymosan-induced paw swelling and pain) for the full spectrum CBD extract. 

For a more in-depth exploration of mechanisms of action of cannabinoids including CBD in cancer, readers are directed to review papers on the subject (e.g., [3,4,92,99]. 

## 6. Evidence of Efficacy of CBD in Management of Cancer and Cancer Treatment-Related Symptoms/Signs

**Figure 1:**
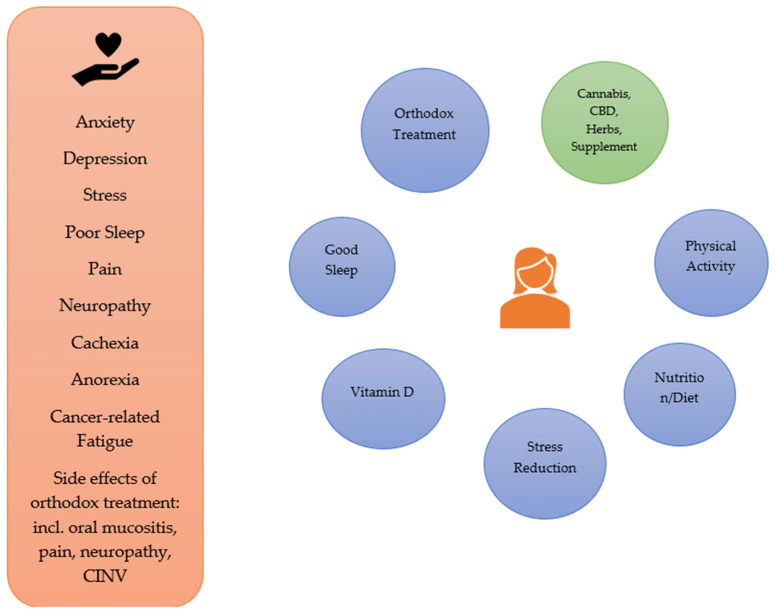
Integrative Approaches to the Symptoms/Signs Associated with Cancer and its Orthodox Treatment.

There are many related signs and symptoms endured by people with living with cancer, due to the cancer itself and/or its treatment. These include stress, anxiety and depression, poor sleep, nausea and vomiting (associated in particular with chemotherapy), pain, neuropathy (e.g., associated with chemotherapy and radiation therapy), oral mucositis (e.g., associated with chemotherapy and head/neck radiation therapy), cancer-related fatigue, cachexia, and anorexia. CBD may have a role as part of an integrative approach to the management of many of these, in conjunction with orthodox treatment as well as consideration of diet, exercise/physical activity, promotion of good sleep, adequate vitamin D levels and stress reduction [122]. See Figure 1.

There are approved cannabinoid drugs on the market that may be used for selected signs/symptoms associated with cancer. For example, the synthetic THC analogue nabilone can be prescribed for chemotherapy-induced nausea and vomiting (CINV), and nabiximols (approximately 1:1 ratio of THC and CBD) is approved in Canada for treatment of cancer-associated pain [123]. 

In this section, we will explore some of the preclinical and clinical evidence for the efficacy of CBD in addressing several of these symptoms and signs. 

### 6.1. Anxiety

Anxiety and depression often co-exist in cancer patients [122]. A large epidemiological study of women diagnosed with breast cancer found that 10.8% had combined anxiety/depression symptoms, 14.9% had only anxiety symptoms, and 2.8% had only depressive symptoms [124]. Endocannabinoid signalling is involved in modulating the stress response, including HPA axis functioning, and can also be altered by stress [125], and anxiety and mood disorders have been found to be associated with a dysfunctional stress response by the HPA axis [40].

Preclinical and clinical evidence indicates that CBD has anxiolytic effects [9]. Several preclinical studies in rodents have demonstrated anxiolytic actions of CBD isolate under conditions of acute administration (e.g., [126,127,128] as well as chronic administration [129,130], though at least one study found no effect of CBD on anxiety [131]. Results of animal studies indicate that the anxiolytic effect of pure CBD follows a bell-shaped dose-response curve, where the anxiolytic effects are found using moderate doses of CBD, but not at lower or higher doses [127,132,133,134]. Note the study mentioned earlier by Gallily and colleagues [121] which reminds us to be careful extrapolating results of CBD isolate to whole plant CBD products. 

There are a few randomised controlled trials that have demonstrated an anxiolytic effect of CBD in healthy volunteers [135,136] and social anxiety disorder (SAD) [137,138] as well as a published case study [139], a retrospective case series in a psychiatric hospital where CBD was used as an adjunct to regular medication [140], and at least three regional brain flow studies in healthy males [141,142] and patients with SAD [143]. 

### 6.2. Depression

Depression is common in cancer patients, affecting up to 25% [144,145]. Prevalence of depression increases with cancer severity and symptoms like pain and fatigue [146]. Some evidence suggests that chronic depression may be associated with an increased risk of cancer [146,147,148] and that depression predicts cancer progression and mortality [146]. Depressive symptomatology has been found to be a consistent psychological predictor of decreased survival time [149]. 

Preclinical evidence indicates that CBD may reduce depressive-like behaviours, via several potential mechanisms of action (see [9]). Several animal studies have shown that CBD can reduce autonomic indices of stress and depression- and anxiety-like behaviours [128,150,151,152,153,154,155,156,157]. In terms of human studies, there are some published case studies which indicate that CBD reduced depressive symptoms [158,159] but no RCTs published in this area yet. 

### 6.3. Sleep Disorders

In newly diagnosed or recently treated cancer patients, the prevalence of sleep disturbances is somewhere between 20% and 75% [160,161,162]. Moreover, insomnia can continue long after treatment: symptoms of insomnia have been found in 23–44% of patients 2–5 years after treatment for cancer [163,164]. It is very important to address poor sleep in cancer sufferers, since insomnia and sleep disturbances can lead to fatigue, mood disturbance including depression, and contribute to immune suppression which affects quality of life and may also impact the course of the disease [165,166]. 

In a survey of 163 cannabis users, 49.7% were using it to treat insomnia and 8.5% to reduce nightmares. This survey found that those with current insomnia and greater sleep latency were significantly more likely to report using strains of cannabis with higher concentrations of CBD [167]. There are a few studies including case reports [139,168], case series [140], and randomized controlled trials [169,170] that indicate that CBD may be efficacious in promoting sleep. Yet, there is some contention in the literature, with some studies suggesting CBD has a stimulating or alerting effect [171,172], and others suggest CBD has a sedating effect [169,170] and one which found no effect in terms of sleepiness [173]. There is a need for research into the potential effects of CBD on sleep in cancer patients, since the benefits of improved sleep would be tremendous. 

### 6.4. Nausea and Vomiting

Chemotherapy-induced nausea and vomiting (CINV) is an undesirable side effect of chemotherapy that can lead to cessation of treatment. Whilst western pharmaceuticals can treat vomiting quite well, treatment of nausea (acute, delayed, anticipatory) is not so effective (. Much of the human research in chemotherapy-induced nausea and vomiting (CINV) has been into synthetic THC such as dronabinol and nabilone, or the combination of CBD and THC (e.g., [174,175]; and systematic reviews: [176,177]. The National Academies of Sciences, Engineering and Medicine 2017 report on cannabis and cannabinoids which used only evidence from systematic reviews and randomised controlled trials (RCTs) concluded that there is conclusive or substantial evidence that oral cannabinoids are effective anti-emetics in the treatment of CINV [178].

Parker reports that animal studies have found CBD can suppress nausea and vomiting within a limited dosage range, with the anti-nausea/emetic effects potentially mediated via indirect activation of somatodentritic 5-HT_1A_ receptors in the dorsal raphe nucleus which reduces release of 5-HT in terminal forebrain areas [40]. Various animal studies indicate that cannabinoid acids (cannabidiolic acid (CBDA) and tetrahydrocannabinolic acid (THCA)) can suppress nausea [179,180,181,182], with CBDA 1000 times more potent than CBD in decreasing acute and anticipatory nausea [181]. In addition, subthreshold doses of CBDA potentiated the anti-nausea effect of Ondansetron (see [182]). More research is needed in humans to ascertain if CBD-predominant products may be effective or not. 

### 6.5. Cachexia

An estimated 20% of cancer deaths are attributed to cachexia [183]. There are several pathways involved, however a key part of the pathomechanism is an inflammatory cascade produced by host and tumour that leads to an imbalance between proinflammatory cytokines (e.g., IL-1, IL-6, interferon γ, and TNF-α and subsequently NF-κB) and anti-inflammatory cytokines [183]. These cytokines act on many different targets (including adipocytes, myocytes, endothelial cells, neurons, bone marrow, and hepatocytes) which eventually lead to weight loss, anorexia, anaemia, depleted lipid stories, severe loss of skeletal muscle protein, metabolic changes, and asthenia (Tamai et al., 2010). There may also be glucose intolerance, insulin resistance, increased fat oxidation, reduced lipogenesis and increased gluconeogenesis that can occur [183].

In general, research into cannabinoids for treatment of cancer cachexia has focused on THC or THC plus CBD (e.g., [184,185,186]. For example, a study in cancer patients found that THC significantly improved pre-meal appetite and proportion of calories consumed as protein compared with placebo [185]. A systematic review that included three studies (n = 592 participants) found that cannabinoids increased appetite, but decreased quality of life which may have been due to the side effects of cannabis. The three studies included one study of THC, and two with THC and combined THC/CBD products [85]. Since CBD has anti-inflammatory properties, one could speculate that (perhaps) it could have a role to play in preventing the imbalance between the pro- and anti-inflammatory cytokines that are part of the pathomechanism of cachexia. More research is needed. 

### 6.6. Cancer-Related Pain and Neuropathy 

Again, much of the research in cancer-related pain/neuropathy and cannabinoids focuses on THC or THC plus CBD, rather than CBD products alone. In an open-label follow-up study of 43 patients experiencing inadequate analgesia despite chronic opioid dosing, where patients were part of a previous three-arm study conducted over two weeks (THC/CBD spray, THC spray, placebo), study participants self-titrated nabiximols (approximate 1:1 ratio CBD:THC) (n = 39) or THC (n = 4). Median duration of treatment was 25 days (2–579 days) for the nabiximols group. The Brief Pain Inventory-Short Form scores for pain severity and worst pain domains both showed improvements, and the quality-of-life questionnaire showed improvements in the domains of pain, fatigue, and insomnia from baseline for the nabiximols treatment [187]. Participants taking nabiximols needed significantly fewer doses of breakthrough pain medication in comparison with placebo, and those who kept using the study medication did not seek to increase the dose or dose of other analgesics over time [187].

In a placebo-controlled double-blind RCT, 263 patients with advanced cancer and opioid-refractory pain were randomised to low, medium, and high doses of nabiximols or placebo. At five weeks follow-up, low and medium dose nabiximols were significantly more effective in reducing average daily pain compared with placebo and only the high dose nabiximols group compared unfavourably with placebo in terms of adverse events [188]. 

In a study assessing comparative effectiveness of THC dominant (n = 52), CBD dominant (n = 19), and mixed (n = 33) in 108 cancer patients, over one month, in general there were no differences between the three treatments in pain intensity (in all three groups there was an improvement from baseline) and no difference between groups in most of the secondary outcome variables measured (only in sleep duration was the THC-dominant treatment significantly superior than the CBD-dominant treatment). There were no differences in the safety profiles between groups [189].

Paclitaxel (PAC) can cause chemotherapy-induced neuropathic pain which can lead to cessation of treatment. Promisingly, CBD has been found to prevent PAC- induced mechanical and thermal sensitivity in mice, and CBD’s protective capacity against PAC-induced neurotoxicity may be mediated in part by the 5-HT_1A_ receptor system [190].

### 6.7. Oral Mucositis

Oral mucositis (OM) can be a common side effect of radiotherapy of the head and neck area (30–70% of patients) and of chemotherapy (40–80% of patients), characterised by ulcers in the oral cavity, and consequent difficulty in eating, weight loss and malnutrition, and susceptibility to infections and poor quality of life [191]. CBD has potential to protect against development of OM given its anti-oxidant, anti-inflammatory and analgesic properties [191,192], though there is a relative lack of research in this area. In a mice model of oral mucositis induced by 5-fluorouracil (5-FU), intraperitoneal administration of CBD was associated with faster resolution of oral lesions and the oral lesions were less severe compared with the positive control (5-FU + mechanical trauma + placebo) (de Freitas Cuba et al., 2020). Results indicated that CBD reduced inflammation, oxidative stress and severity of lesions, facilitating tissue repair [192]. Although not specifically associated with OM, research in the field of dentistry in a rat model of periodontitis may be of relevance to OM. A study in rats indicated that CBD treatment reduced alveolar bone loss, lowered NF-kB and was associated with a decreased neutrophil migration, and decreased production of pro-inflammatory cytokines interleukin-1β and TNF-α [193]. OM is definitely an area for researching the potential effects of CBD, particularly as there is little in the pharmaceutical line to treat it successfully. 

### 6.8. Palliative Care

Where cannabis products, including those containing THC may be of particular value is at end of life, in palliative care. An Israeli study analysed data for 2970 cancer patients treated with medicinal cannabis between 2015 and 2017. The most frequent types of cancer where breast, lung, pancreatic, and colorectal (51.2% of study participants were at stage 4). Mean age was 59.5 +/− 16 years. The main symptoms that participants were using cannabis for were sleep problems (78.4%), pain (77.7%, median intensity 8/10), nausea (64.6%), and lack of appetite (48.9%). At the six-month follow-up, 24.9% of patients had died, and 18.8% had stopped treatment. Of the remaining, 60.6% (n = 1211) responded to the questionnaire. Of this group, 95.9% reported an improvement in their medical condition, 3.7% reported no change, and 0.3% reported a deterioration. Symptoms which were most improved were: nausea and vomiting (91.0%), sleep disorders (87.5%), restlessness (87.5%), anxiety and depression (84.2%), pruritus (82.1%), and headaches (81.4%). In addition, 35.1% reported a decrease in their regular medication consumption, mainly in the following drug categories: other analgesics and antipyretics, hypnotics and sedatives, corticosteroids and opioids. At least one side effect was reported at 6 months follow-up by 30.1% of patients (n = 362) with the most common being: dizziness (n = 96, 8%), dry mouth (n = 88, 7.3%), sleepiness (n = 40, 3.3%), and psychoactive effects (n = 34, 2.8%). The authors concluded that cannabis used for palliative care in patients was a well-tolerated, effective, and safe option to help manage cancer-related symptoms [194]. 

## 7. Combining CBD with Orthodox Cancer Treatment

There is increasing evidence that CBD can synergise with various chemotherapeutic agents to increase their efficacy. For example, CBD has been found to sensitize cancer cells to cisplatin and significantly increase cisplatin-mediated apoptosis, suggesting a potential adjuvant role [25]. Ferro and colleagues [120] confirmed that GPR-55 signalling promotes the proliferation of pancreatic ductal adenocarcinoma cells (PDAC) and growth of tumours, and that GPR-55 inhibition reduced cancer progression and significantly improved survival in a transgenic PDAC mice model. They found that CBD enhanced the effects of gemcitabine: mice treated with a combination of CBD and gemcitabine survived almost three times longer than mice treated with gemcitabine alone (Ferro et al., 2018). The combination also opposed mechanisms involved in the development of gemcitabine resistance, both in vitro and in vivo [120]. This gives some hope for a form of cancer with a very low survival rate. 

Other studies have found that the combination of CBD and THC is able to work synergistically with chemotherapeutic agents. A combination of CBD and THC reduced multiple myeloma (MM) cell migration by downregulating the expressions of chemokine receptor CXCR4 and the CD147 plasma membrane glycoprotein, and the CBD-THC combination in conjunction with the immuno-proteasome inhibitor carfilzomib acted synergistically to increase MM cell death and inhibit cell migration [195]. CBD alone and in combination with the proteasome inhibitor bortezomib was found to strongly inhibit growth, arrest cell cycle progression, and induce death of multiple myeloma cells by regulating the ERK, AKT and NF-κB pathways with major effects occurring in TRPV2+ cells (Morelli et al., 2014). TRPV2, a member of the transient receptor potential (TRP) cation channel family, is involved in the regulation of tumour growth, progression, invasion, and angiogenesis (Morelli et al., 2014). Studies in glioma cell lines have demonstrated that CBD triggered TRPV2-dependent Ca2+ influx, increasing uptake of chemotherapeutic drugs temozolomide, carmustine, and doxorubicin, and synergising with the drugs to induce apoptosis of glioma cells (i.e., the pro-apoptotic effect was greater than the drugs alone) with no such effect in normal human astrocytes [196]. 

In a leukemia cell line study when CBD was paired with either THC or another phytocannabinoid cannabigerol (CBG), the cytotoxic effect (measured by the IC_50_) in acute lymphocytic leukaemia (CEM) cells was stronger than when each was used separately. Moreover, CBD and THC combined synergistically with common anti-leukemia drugs, sensitising leukemia cells to the cytotoxic effects of the drugs and allowing the doses of these drugs to be reduced substantially whilst remaining efficacious. This study also found that the timing of administration was important: using cannabinoids after chemotherapy resulted in greater apoptosis induction, whilst if used prior to chemotherapy, there was less induction of apoptosis [197]. 

Although not discussed in this paper, there is much research to suggest that THC has many anti-cancer activities and can enhance chemotherapeutic drugs. However, a difficulty associated with high doses of THC are the undesirable intoxicating effects. THC has been found to enhance the effects of temozolomide in mice glioma xenografts, including in temozolomide-resistant tumours (the combination enhanced autophagy), and without toxic side effects [198]. This study also found that submaximal doses of THC combined with CBD greatly decreased the viability of human glioma cell lines and primary cultures of human glioma cells, and reduced the growth of U87MG cell-derived subcutaneous xenografts to a greater degree than either alone. They further found that the combination of submaximal doses of CBD and THC reduced tumour growth to a similar extent as an effective dose of THC, suggesting that CBD’s ability to enhance THC’s antitumoral action could allow the reduction of the amount of THC and thus reduce the undesirable psychoactive effects associated with THC [198]. 

### 7.1. Potential Neuroprotective Effects

Given CBD’s neuroprotective actions, one can surmise that it may be useful in protecting normal neural tissue from radiation and chemotherapy damage. Brain tumour treatment, including glioblastoma, utilises external beam radiation therapy alone or in combination with chemotherapy (e.g., temozolomide), however this treatment can cause severe damage to normal brain tissues (especially adult neurons and endothelial cells). One study found that CBD enhanced the effects of ϒ-radiation, opening up the possibility that lower doses of radiation might be able to be used in combination with CBD and THC [108]. As mentioned previously, a mice study found that CBD is protective against paclitaxel-induced neurotoxicity and in a separate experiment, found that CBD did not reduce the paclitaxel-induced inhibition of breast cancer cell viability [190]. Boullon and colleagues’ review suggests the potential for cannabinoids to address chemotherapy-induced cognitive impairment, given evidence of cannabinoids for the modulation of neuroinflammation and oxidative stress in different pathologies with a similar cognitive phenotype to CICI [199]. 

### 7.2. Potential Organ-Protective Effects

Other animal studies suggest protective effects of CBD on various organs against chemotherapeutic toxicity including a cardioprotective effect of CBD against doxorubicin-induced cardiac injury [200] and nephroprotective effects against cisplatin [201]. CBD reduced cisplatin-induced nephropathy by decreasing oxidative/nitrosative stress, inflammation and cell death in a mice model of cisplatin-induced nephropathy, improving renal function [201].

### 7.3. Cautions

However, some studies do suggest caution in relation to cannabis and immunotherapy. A retrospective study in cancer patients (Stage 4 non-small cell lung cancer, clear cell renal cell carcinoma and advanced melanoma) treated with nivolumab immunotherapy found that those who used cannabis had a decreased response rate (RR) compared with those who did not use cannabis: 37.5% and 15.9% RR in nivolumab alone group and nivolumab-cannabis group, respectively (*p* = 0.016, odds ratio = 3.13, 95% confidence interval 1.24–8.1), though there was no significant difference in overall survival and progression-free survival. THC and CBD percentages did not affect RR in any group [202]. 

The potential for drug-CBD interactions is also important more broadly. According to the World Health Organization there is potential for CBD to be associated with drug interactions through inhibition of some cytochrome P450 enzymes, but it is not yet clear whether these effects occur at physiological concentrations [17]. Drug-CBD interactions can be pharmacokinetic or pharmacodynamic in nature [203,204].

More research is needed in the area of immunotherapy drugs and potential interactions with CBD, in particular as CBD may often be preferred due to its lack of potentially intoxicating effects. 

## 8. Potential Barriers to Use in Oncology 

Quite often barriers to the incorporation of therapies outside of orthodox medicine is simply lack of knowledge of medical practitioners. In the opinion of the author, it is no different with medicinal cannabis and CBD, and of course, there is the added stigma attached to cannabis that still influences many. 

A survey of 103 oncology health care practitioners (HCPs) which included oncology nurses, radiation therapists, and pharmacists, found that 69% had spoken to a patient about cannabis in the preceding month, and 84% felt they lacked enough knowledge about cannabis to make any recommendations. Commonly reported barriers cited included monitoring the patient’s use of cannabis (54%), prescribing an accurate dose (61%) or strain (53%), and having insufficient research (50%). However more than 50% of HCPs surveyed indicated interest in receiving more information or training about cannabis use in oncology [205]. In the survey at the Fred Hutchinson Cancer Centre mentioned previously, nearly all the cancer patients surveyed preferred to get information from their cancer team (74%) but less than 15% actually received any information about cannabis from the cancer doctor or nurse, with most receiving information from friends/family, newspapers, websites, other cancer patients, and other sources (and more than one-third reported not receiving any information) [1]. The online survey of breast cancer sufferers in the US mentioned previously found that the majority of patients (61%) did not discuss cannabis use with their doctor. This survey also revealed that the internet and family/friends were the most common sources of information about cannabis [2].

Other barriers to use in oncology include the fact that cannabis products are simply not readily available in the orthodox medical system, a lack of standardisation of CBD and cannabis products, variable quality of products, and in the US, the fact that cannabis is still a Schedule 1 drug. There is also likely to be a paucity of pharmacists knowledgeable in the dispensing of cannabinoid medicines in many countries.

Nonetheless, in order to better serve patients (as that is the role of the clinician ultimately), it behoves the clinician to at least become better informed about medicinal cannabis, so that they can talk informatively with patients.

## 9. Conclusions

CBD is a key component of the cannabis plant and does not have the potentially intoxicating effect associated with THC. CBD products are not ‘one thing’—there are many different CBD products, and they vary not only in terms of route of delivery, but also in terms of the amount of CBD, presence of other phytocannabinoids, terpenes, flavonoids, and other plant nutrients (which are likely to add to the overall therapeutic effect). Whole plant products are likely to work differently to CBD isolates. There is preclinical evidence that CBD may address several of the pathways underpinning cancer, and there is preclinical and some clinical evidence that CBD may be effective in treating several of the symptoms and signs associated with the disease and its orthodox treatment.

Cancer patients are using cannabis products. It is the responsibility of healthcare practitioners, including oncologists, to be informed of the growing evidence base associated with cannabis and CBD, and to keep an open mind as this plant medicine may well have an important role to play in the integrative care and journey of the cancer patient.

## 10. Declarations

Professor O’Brien works as Chief Scientific Officer for Releaf Group Ltd., St Kilda, Australia, has conducted consultancies to several cannabis companies in the past, and owns shares in cannabis companies. She is also the founder of the International College of Cannabinoid Medicine (iccm.co), London, UK, an online education portal for healthcare practitioners focused on the evidence-base of medicinal cannabis.

## Figures and Tables

**Table 1:** Therapeutic Actions of CBD (adapted from [9]).

analgesic anti-nausea anti-emetic anxiolytic antidepressant anti-psychotic anti-convulsant/anti-epileptic anti-asthmatic	immune-modulatory antioxidant anti-inflammatory antibiotic, anti-bacterial neuroprotective anti-cancer and anti-tumoral [17,18,23,24,25,26,27]

**Table 2:** Location of CB1 and CB2 Receptors (adapted from [9]).

CB1 Receptors	CB2 Receptors
**Abundant in the Central Nervous System:** Brain nerves/neurons in particular; also present in cerebral blood vessels; also to some extent in microglia, astrocytes; spinal cord **Also present in:** Peripheral Nervous System: sympathetic nerve terminals, trigeminal ganglion, dorsal root ganglion, dermic endings of primary sensory neurons; neurons of parasympathetic nervous system Blood, Tissues, Immune Cells: adipose tissue (white, brown), connective tissue, fascia, fibroblasts, skeletal muscle, bone (osteoclasts, osteoblasts); smooth muscle (vascular and visceral); blood vessels, vascular endothelial cells, blood (leukocytes), vascular smooth muscle cells; immune cells including macrophages, mast cells Organs & Glands: skin, GI tract, eye, liver, heart, kidney, bladder, adrenal gland, spleen, tonsils. lung, endocrine glands (e.g., thyroid, adrenals, pituitary gland), exocrine glands, reproductive organs: male (testes) and female (uterus, ovaries), placenta	**Highly Concentrated in Cells/Tissues/Organs of Immune System:** Monocytes, macrophages, CD4+ and CD8+ T Cells, B-cells, NK cells, neutrophils, mast cells; spleen, tonsils, thymus **Also present in:** CNS (present in lower levels in CNS): cell bodies and dendrites of central neurons; cortex, brainstem, cerebellum, striatum, hippocampus, amygdala, retina, neuronal, glial (astrocytes, microglia) and endothelial cells of brain; Spinal Cord and Dorsal Root Ganglia Blood, Tissues, Cells: various human tumours, adipocytes, leucocytes, bone marrow; bone (osteoclasts, osteoblasts, osteocytes), muscle cells, human vascular smooth muscle, endothelial cells Organs: skin, GI tract, liver, heart, pancreas, spleen, lung, kidneys, bladder, reproductive organs & cells (e.g., ovary); placenta

[32,38,40,42,46,47,48,50,51,52,53,54,55,56,57,58,59,60,61,62,63,64,65,66,67,68,69,70,71,72,73,74,75,76,77,78,79,80,81,82,83].
